# Supplementary figures and images for: Revealing key regulators of neutrophil function during inflammation by re-analysing single-cell RNA-seq
Source: PLoS One. 2022 Oct 21;17(10):e0276460. doi: 10.1371/journal.pone.0276460 (PMC9586406; doi:10.1371/journal.pone.0276460)

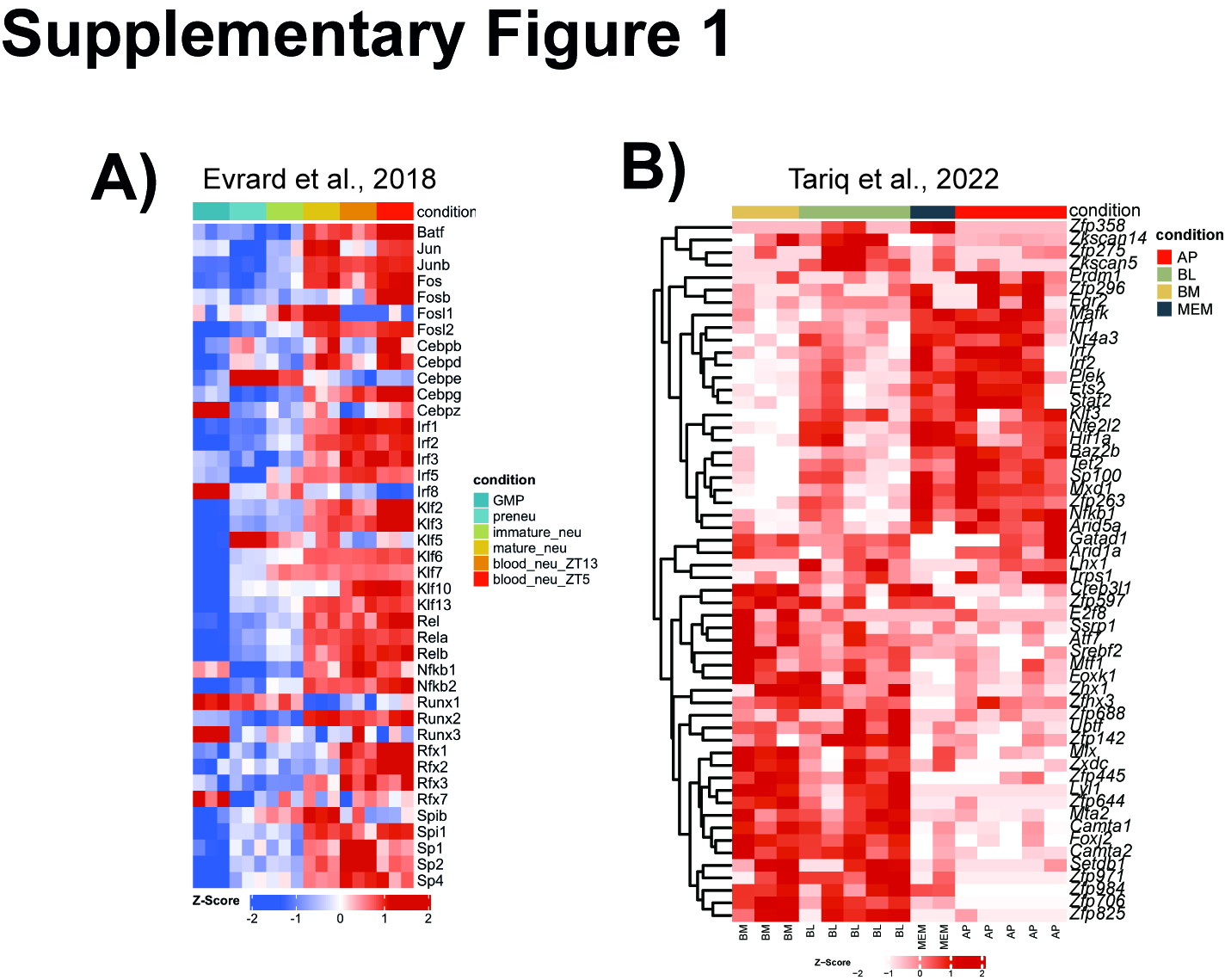

Supplement: S1 Fig — (A) Heatmap showing row-scaled expression of the signature TFs in bulk RNA-seq analysis of neutrophil populations in the bone marrow and blood (Evrard et al. 2018, Immunity). (B) Heatmap showing the scaled expression of signature genes for each bone marrow neutrophil subpopulation from the bone marrow(BM), blood(BL), air pouch membrane(MEM) and exudate(AP), coloured by the average expression of each gene in each cluster scaled across all clusters (Tariq et al. 2022, Nature Immunology). (TIF) [file pone.0276460.s001.tif]
